# Energy landscape for the insertion of amphiphilic nanoparticles into lipid membranes: A computational study

**DOI:** 10.1371/journal.pone.0209492

**Published:** 2019-01-09

**Authors:** Reid C. Van Lehn, Alfredo Alexander-Katz

**Affiliations:** 1 Department of Chemical and Biological Engineering, University of Wisconsin-Madison, Madison, WI, United States of America; 2 Department of Materials Science and Engineering, Massachusetts Institute of Technology, Cambridge, MA, United States of America; University of Waterloo, CANADA

## Abstract

Amphiphilic, monolayer-protected gold nanoparticles (NPs) have been shown to enter cells via a non-endocytic, non-disruptive pathway that could be valuable for biomedical applications. The same NPs were also found to insert into a series of model cell membranes as a precursor to cellular uptake, but the insertion mechanism remains unclear. Previous simulations have demonstrated that an amphiphilic NP can insert into a single leaflet of a planar lipid bilayer, but in this configuration all charged end groups are localized to one side of the bilayer and it is unknown if further insertion is thermodynamically favorable. Here, we use atomistic molecular dynamics simulations to show that an amphiphilic NP can reach the bilayer midplane non-disruptively if charged ligands iteratively “flip” across the bilayer. Ligand flipping is a favorable process that relaxes bilayer curvature, decreases the nonpolar solvent-accessible surface area of the NP monolayer, and increases attractive ligand-lipid electrostatic interactions. Analysis of end group hydration further indicates that iterative ligand flipping can occur on experimentally relevant timescales. Supported by these results, we present a complete energy landscape for the non-disruptive insertion of amphiphilic NPs into lipid bilayers. These findings will help guide the design of NPs to enhance bilayer insertion and non-endocytic cellular uptake, and also provide physical insight into a possible pathway for the translocation of charged biomacromolecules.

## Introduction

Functionalized nanoparticles (NPs) have emerged as versatile materials with physicochemical properties that can be tuned to mimic biological macromolecules, facilitating their use in biomedical applications including drug delivery, bioimaging, and biosensing [1–3]. There is particular interest in understanding how the interactions of NPs with biological membranes can be tailored to achieve efficient cellular uptake [4, 5]. Because the hydrophobic core of the lipid bilayer acts as a barrier to the passive diffusion of hydrophilic molecules, the transport of water-soluble NPs into cells typically occurs via endocytosis. In this process, the membrane wraps around an extracellular NP and pinches off into an intracellular endosome without the NP accessing the cytosol [6]. Recently, small gold NPs (with core diameters < 10 nm) protected by multicomponent alkanethiol monolayers were instead observed to enter cells with endocytosis inhibited [7, 8]. The monolayers contained two components—1-mercaptoundecanesulfonate (MUS), which is anionic, and octanethiol (OT), which is nonpolar—to yield anionic, amphiphilic surface properties. Internalization did not trigger the leakage of a membrane impermeable dye [7], indicating that cell entry does not disrupt the bilayer. By undergoing this novel non-endocytic mode of uptake, these amphiphilic MUS:OT NPs offer significant advantages relative to other NPs; for example, cationic NPs may induce cytotoxic membrane pores [9], while NPs taken up by endocytosis may degrade into toxic byproducts [10]. Early studies have used MUS:OT NPs to deliver siRNA [11] and enhance radiotherapy [12], demonstrating their potential utility for biomedical applications. However, the mechanism of non-endocytic, non-disruptive uptake remains poorly understood, hindering the ability to rationally engineer NP surface properties.

To gain insight into the uptake mechanism, experimental studies have examined the interactions of the same MUS:OT NPs with model cell membranes. Surprisingly, the NPs appeared to insert into the membranes of several model systems, including multilamellar lipid vesicles [8], lipid vesicles with biomimetic compositions [13, 14], black lipid membranes [15], and supported lipid bilayers [16], despite the high surface charge density that should inhibit contact with the hydrophobic bilayer core. Moreover, only the NPs with core sizes and ligand ratios that enabled favorable insertion into model bilayers entered cells when endocytosis was inhibited [8]. These results identify bilayer insertion as a critical process that must be understood to identify NP components that maximize cellular internalization, but these experiments alone are unable to provide molecular insight into the mechanism of bilayer insertion.

To complement experimental measurements, computational methods have emerged as a powerful tool to resolve the molecular details of NP-bilayer interactions. For example, molecular simulations have been used to investigate the binding of NPs to single and multicomponent bilayers [17–21], the wrapping of the bilayer around bound NPs [22–26], the insertion of amphiphilic NPs into the bilayer [16, 27–29], the translocation of NPs across the bilayer [30–36], and the disruption of the bilayer induced by NPs [37–39]. These examples represent only a subset of simulation approaches, with more examples detailed in recent review articles covering this field [40–45]. In our prior work, molecular dynamics simulations were used to study the interactions of MUS:OT NPs with single-component lipid bilayers [16, 29, 36]. Unbiased simulations confirmed that a MUS:OT NP spontaneously inserts into the bilayer when placed near the high-curvature edge of a bilayer ribbon, a simulation construct that mimics the edge of a large bilayer defect [16]. Insertion is triggered by contact between the NP monolayer and a solvent-exposed lipid tail protrusion and is driven by ligand deformations that shield hydrophobic ligand backbones within the bilayer core while retaining charged end groups in solution [16]. The NP ultimately reaches a membrane-spanning configuration with charged end groups equally distributed on both sides of the bilayer (S1 Fig). A MUS:OT NP can also insert into a single leaflet of a planar, defect-free bilayer if contact with a protruding lipid occurs [29], although such protrusions are rare in simulations [46]. Insertion into a planar bilayer is consistent with experiments that show no evidence of large bilayer defects prior to or during NP insertion [7, 8]. However, charged ligand end groups must cross the hydrophobic bilayer core in order to reach a membrane-spanning configuration in a defect-free bilayer (S1 Fig). While the ribbon simulations suggest that a membrane-spanning configuration is preferred, it is unknown if this configuration is thermodynamically favorable in a planar bilayer or if it can be reached on experimental timescales (estimated as seconds for cellular internalization; private communication with F. Stellacci). Because a membrane-spanning configuration may allow the NP to access the cytosol in a cellular system, it is of particular interest to understand the molecular pathway to this configuration from an initial configuration in which the NP is inserted into a single bilayer leaflet.

In this work, we use atomistic molecular dynamics simulations to investigate the hypothesis that an amphiphilic MUS:OT NP reaches a membrane-spanning configuration in a planar, defect-free lipid bilayer by “flipping” charged ligands across the bilayer core. In this flipping process, a charged MUS ligand end group crosses from one bilayer leaflet to the other in analogy to lipid flip-flop [47]. Starting from a configuration in which the NP is inserted in the upper leaflet of the bilayer, we apply biasing forces to flip charged ligands across the bilayer midplane. This procedure allows us to analyze a series of intermediate states in which the distribution of ligands on either side of the membrane varies, representing possible states encountered during bilayer insertion that have not been analyzed in prior studies [16, 28, 29, 36, 48]. We show that sequential ligand flipping relaxes bilayer curvature, decreases the nonpolar solvent-exposed surface area of the NP, and increases favorable interactions between charged ligand end groups and lipid head groups, all driving forces that prefer a fully inserted, membrane-spanning configuration. We further find that at least one charged ligand end group is dehydrated for each ligand distribution along the insertion pathway, enabling insertion on experimentally accessibly timescales. Building on prior findings, these results support a complete energy landscape for NP-bilayer insertion that may be used to guide the design of nanomaterials that enter cells via a non-disruptive, non-endocytic uptake mechanism.

## Materials and methods

**Fig 1:**
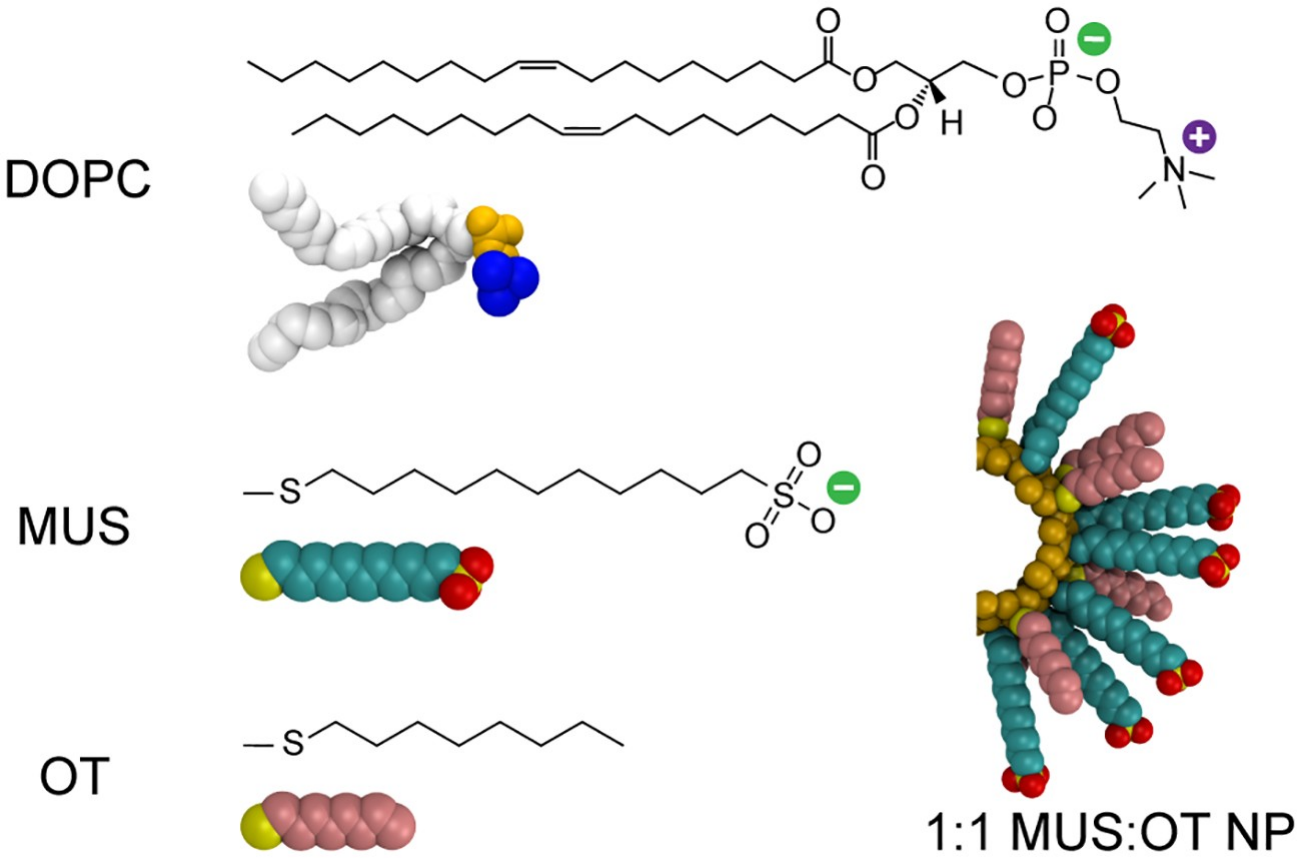
Simulation snapshots and corresponding chemical structures of the system components. The bilayer is composed of the lipid DOPC, which has two hydrophobic tails (white) and a zwitterionic head group (choline in blue, phosphate in yellow). Two ligands, MUS and OT, are grafted to a gold NP core in a 1:1 ratio. MUS has a hydrophobic backbone (cyan) and anionic sulfonate end group (yellow/red), while OT is purely hydrophobic (pink). Both ligands terminate in sulfur atoms bonded to the gold surface (yellow).

Atomistic molecular dynamics simulations are performed to model the interaction of an amphiphilic MUS:OT NP with a dioleoylphosphatidylcholine (DOPC) bilayer. Following previous work [16, 29, 36, 49, 50], the NP gold core is modeled as a rigid, hollow sphere with a diameter of 2 nm. The mass of missing gold atoms is redistributed to the atoms in the shell. 58 ligands are grafted to the gold core, corresponding to a surface density of 4.62 ligands/nm^2^ [18]. MUS has an eleven-carbon alkane backbone that is end-functionalized with an anionic sulfonate moiety, while OT has an eight-carbon alkane backbone (Fig 1). All MUS end groups are assumed to be fully dissociated at physiological pH. Both ligands terminate in sulfur atoms that are fixed to the gold core to represent the thiol bond and are assumed to be immobile with respect to the gold surface over the simulation timescale [51]. Ligands are grafted to the gold surface in a 1:1 MUS:OT ratio to mirror prior computational and experimental studies [7, 8, 29, 36]. All system components are modeled using the GROMOS 54a7 united-atom force field [52, 53]. The Simple Point Charge water model is used to match the parameterization of GROMOS [52]. Parameters for gold and sulfonate are both taken from prior work [49]. Additional details on the parameterization are included in S1 Text.

**Fig 2:**
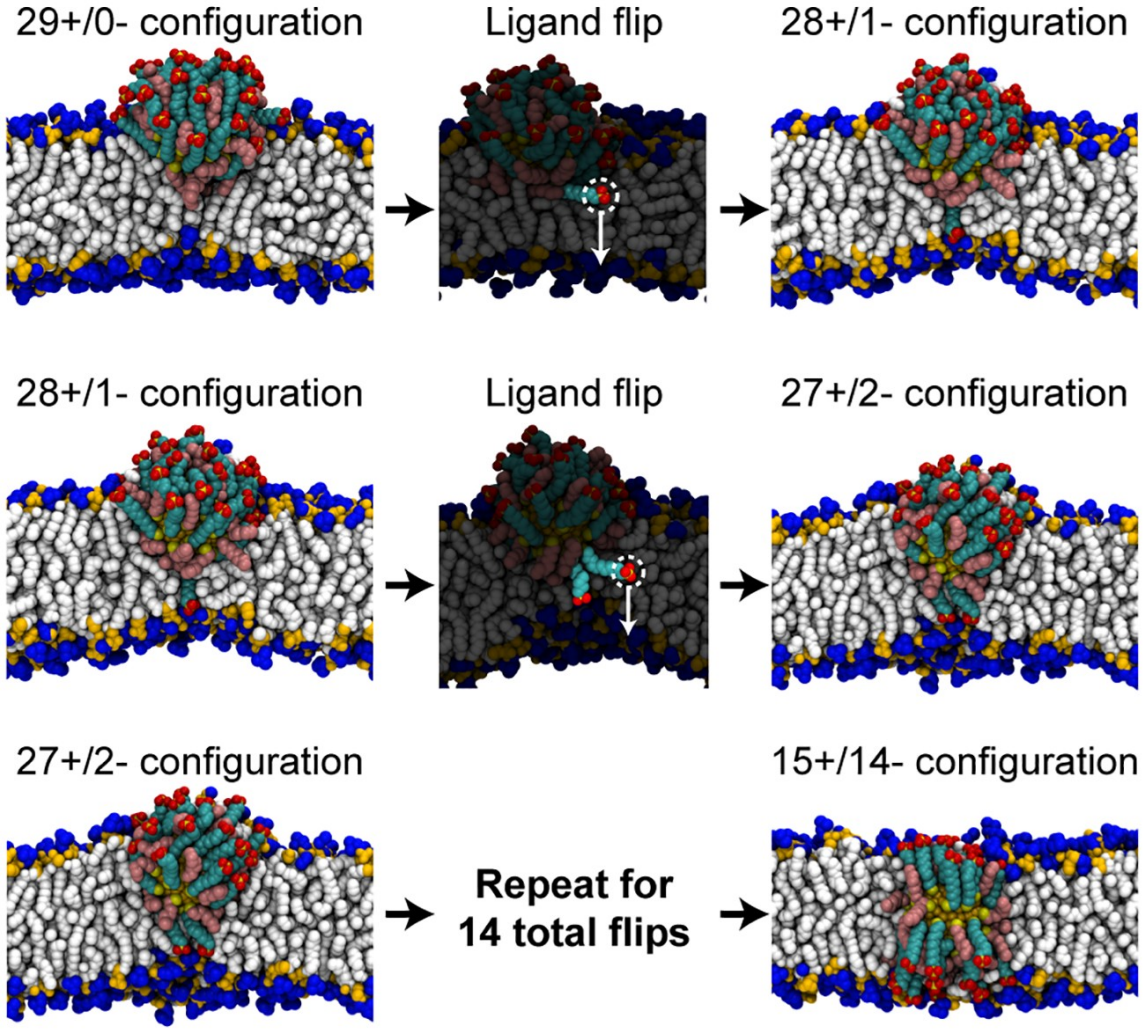
Workflow for flipping charged MUS ligands across a lipid bilayer. The initial configuration (taken from previous work [29]) has 29 MUS ligands positioned above the bilayer midplane and 0 MUS ligands below the midplane (29+/0-). A single MUS ligand (highlighted) is then flipped across the bilayer by applying a biasing potential to obtain the 28+/1- configuration. Water molecules and ions are included in the simulations but are omitted from the snapshots. MUS ligands are then iteratively flipped to generate different distributions of MUS end groups ranging from 29+/0- to 15+/14-. Additional details on this process are included in S1 Text.

Different distributions of MUS ligands with respect to the bilayer are generated by sequentially flipping MUS ligands from the upper leaflet of the bilayer to the lower leaflet of the bilayer, as summarized in Fig 2. Briefly, in this workflow an individual MUS ligand is selected and the distance between the center-of-mass of its sulfonate end group and the bilayer midplane, projected along the z-axis, is calculated; this quantity is defined as *d*_*z*_. A harmonic biasing potential with a spring constant of 3000 kJ/mol/nm^2^ is then applied to the selected ligand’s sulfonate group to decrease *d*_*z*_ and thus flip the ligand across the bilayer over a 1.5 ns time interval. Complete details on this procedure are provided in S1 Text. After flipping, the system is then equilibrated for 20 ns. During this equilibration, *d*_*z*_ is calculated for each MUS ligand. The MUS ligand with the smallest positive average value of *d*_*z*_ is then selected to be flipped next because lower values of *d*_*z*_ correspond to ligands closer to the flipping transition state based on prior free energy calculations [36]. The flipping workflow is initialized from a configuration obtained from Ref. 29 in which a NP is inserted in the upper leaflet with all 29 MUS ligands located above the bilayer midplane (Fig 2). Simulations are initiated from this configuration to avoid reproducing lengthy simulations in which the MUS:OT NP was observed to insert into the upper leaflet from an initial state in solution (detailed in a prior study [29]). This configuration will be referred to as the 29+/0- configuration, with the nomenclature reflecting the number of MUS ligands above (+) and below (-) the bilayer midplane. The flipping process is repeated 14 times to generate a series of MUS ligand distributions ranging from 29+/0- to 15+/14-.

Importantly, flipping is performed for NPs inserted into bilayer ribbons. A bilayer ribbon only spans the simulation box in one dimension. Two edges of the ribbon are exposed to solvent to allow lipids to freely redistribute between leaflets in response to the change in MUS ligand distribution, conserving the preferred area per lipid in each bilayer leaflet to prevent lateral stresses related to incommensurate areas [54]. After each flip, the NP-ribbon systems are converted to conventional box-spanning bilayers (see S1 Text, S2 and S3 Figs) to reduce the system size and to eliminate any potential effects of the free ribbon edge. The final system for every ligand distribution contains 334 lipids, 21,418 water molecules, 89 Na^+^ ions, and 60 Cl^−^ ions to maintain an electroneutral 150 mM NaCl concentration. Each box-spanning configuration is equilibrated for 50 ns and sampled for an additional 120 ns. All results presented below are calculated using the box-spanning bilayers; the bilayer ribbons are only used for system preparation. A detailed description of the flipping workflow is included in S1 Text.

Molecular dynamics is performed using a leap-frog integrator with a timestep of 2 fs. Electrostatic interactions are calculated using the smooth Particle-Mesh Ewald (PME) method [55] with a real space cutoff of 1.0 nm, a grid spacing of 0.12 nm, and fourth-order interpolation. Non-bonded interactions are calculated with a neighbor-list cutoff of 1.0 nm and a Lennard-Jones cutoff of 1.0 nm. These parameters are chosen in accordance with an evaluation of GROMOS 54a7 using PME [53]. All bonds are constrained with the LINCS algorithm [56]. The simulation temperature is set to 310 K using a velocity-rescale thermostat with a coupling time of 0.1 ps. The simulation pressure is set to 1 bar and is controlled using a Parrinello-Rahman barostat with a coupling time of 2 ps and an isothermal compressibility of 4.5 ×10^−5^ bar^−1^. The method used to scale the simulation box vectors is chosen to reflect the symmetry of specific simulation conditions as summarized in S1 Text. All simulations are performed using version 4.6.7 of the Gromacs simulation package [57]. The error of each sampled quantity is estimated with block averaging using the Gromacs program *g_analyze* [58].

## Results

### MUS flipping allows a NP to non-disruptively reach the bilayer midplane

**Fig 3:**
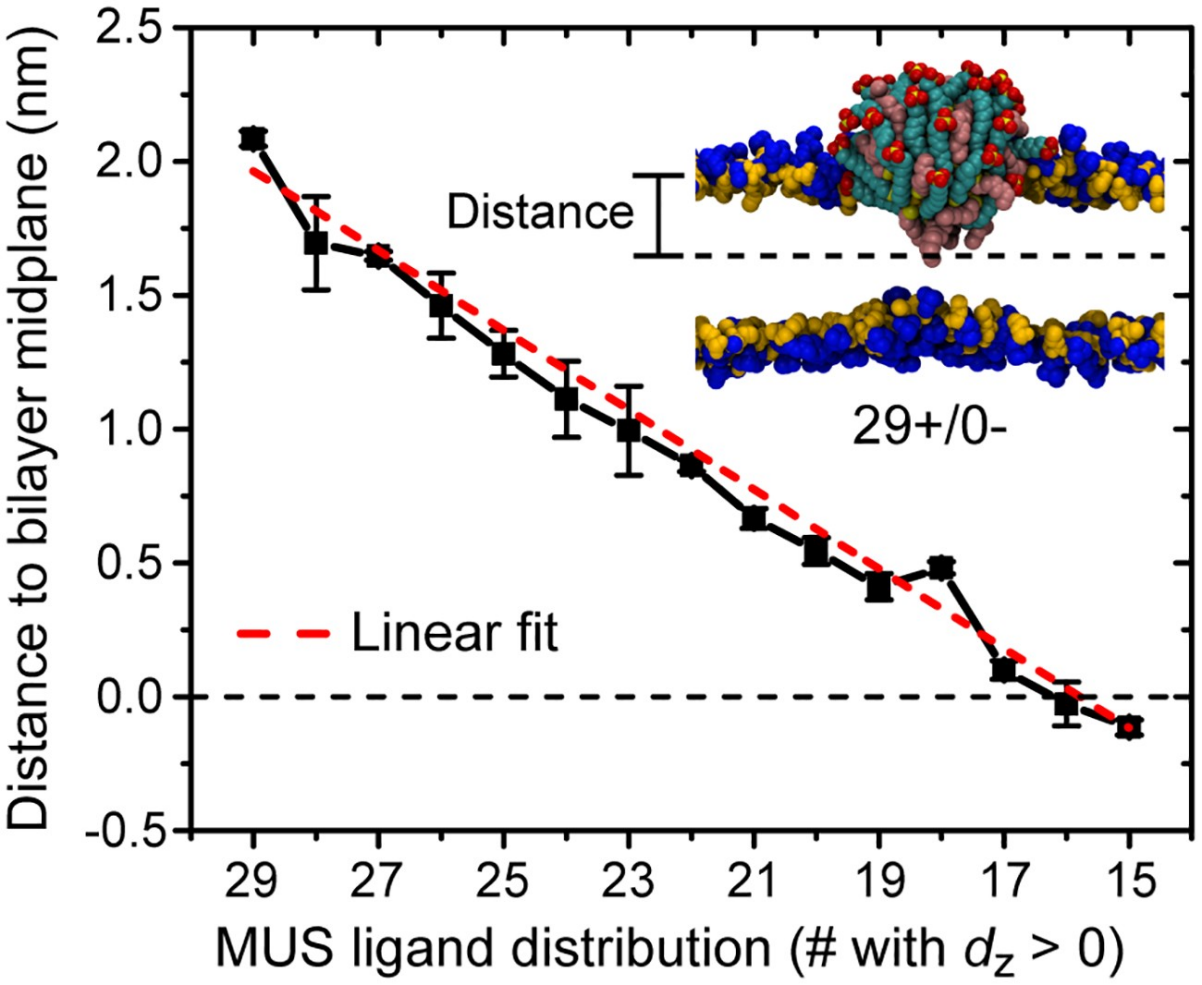
Distance between the center of the NP gold core and the bilayer midplane as a function of the distribution of MUS ligands. The distance is projected along the z-axis of the system as illustrated in the accompanying snapshot; the horizontal dashed line indicates the bilayer midplane. The dashed red line is a linear fit to the data.

Flipping MUS ligands across the bilayer from a 29+/0- configuration, in which all 29 MUS ligands are positioned above the bilayer midplane and the NP core is localized to the upper leaflet of the bilayer [29], to a 15+/14- configuration allows the NP to reach a fully inserted configuration in which the NP gold core is located at approximately the bilayer midplane. Fig 3 shows that the distance between the center of the NP gold core and the bilayer midplane decreases approximately linearly as MUS ligands flip. No significant bilayer disruption, such as pore formation, is induced by any MUS ligand distribution. Spontaneous, unbiased MUS flipping is not observed, indicating that each MUS distribution is metastable within the 170 ns simulation runtime and that the bilayer permeability is not significantly increased. While a prior study has shown that multiple charged arginine molecules can cooperatively translocate across a bilayer [59], such concerted flipping events are not observed here. Finally, lipid flip-flop is not observed for any ligand distribution or during any of the ligand flipping processes, although lipids are able to transfer from the upper to lower leaflet during system preparation (see S1 Text). The data thus support a pathway in which a series of individual MUS ligand flips connect the partially inserted state to the fully inserted configuration predicted previously [8, 16, 48, 60] while maintaining the barrier properties of the bilayer.

**Fig 4:**
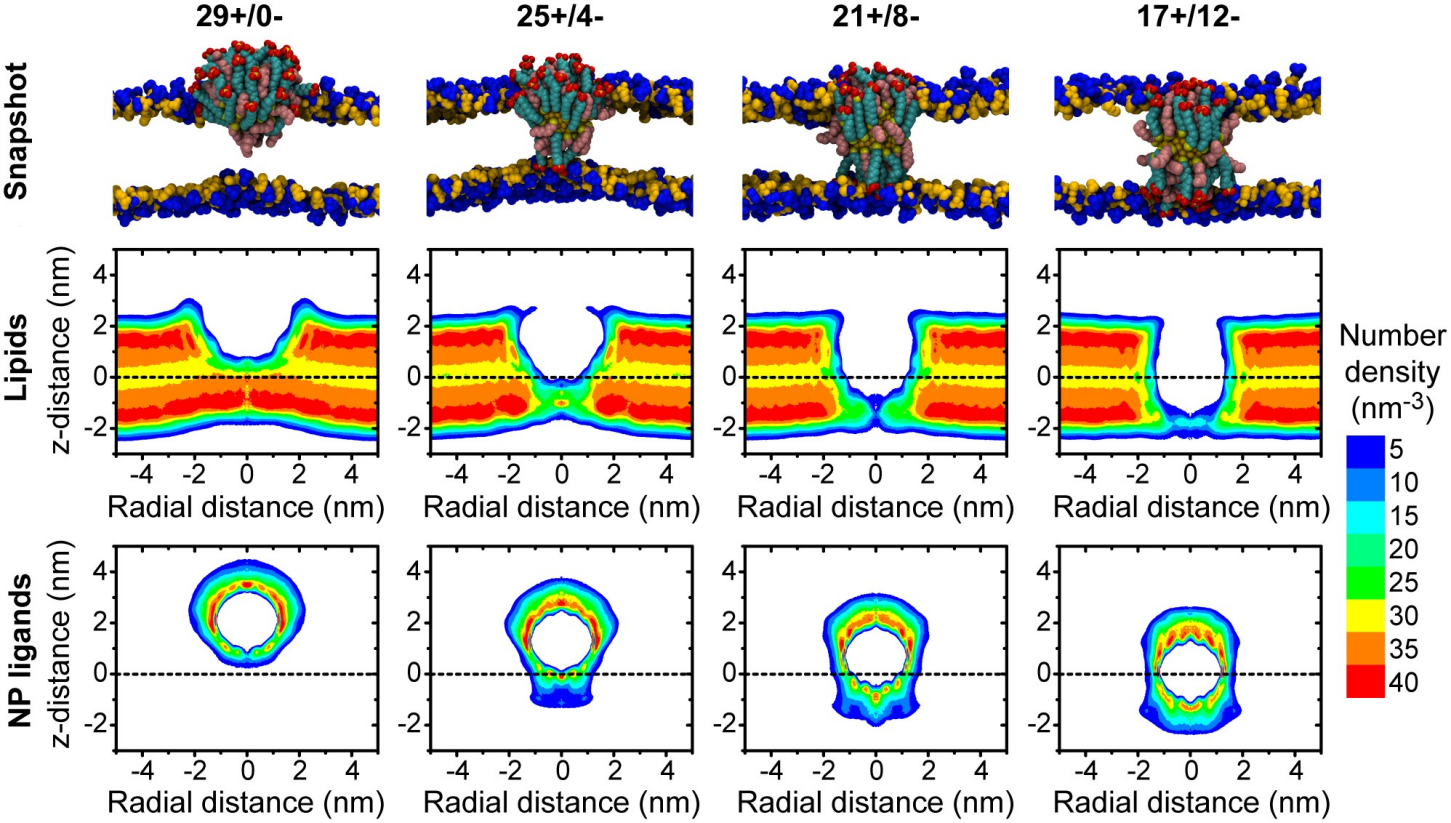
Configurations of lipids and NP ligands for different MUS ligand distributions. The top row presents representative simulation snapshots of the NP and lipid head groups for four MUS distributions; lipid tail groups, water molecules, and ions are not shown. The middle row presents the time-averaged number density of all lipid atoms. The bottom row presents the time-averaged number density of all ligand atoms, including both MUS and OT ligands. Due to the cylindrical symmetry of the system, number densities are plotted as a function of the distance in the x-y plane from the center of the NP gold core (radial distance) and the distance along the z-axis from the bilayer midplane (z-distance). Positive and negative values of the radial distance are equivalent and negative values are included only as a visual aid.

To further illustrate the conformational changes associated with NP insertion, Fig 4 presents the number densities and simulation snapshots of both lipids and NP ligands for four representative MUS ligand distributions. Comparing the density profiles shows that hydrophobic ligand backbones maximize contact with the hydrophobic lipid core. Maintaining this interface both slightly thins the bilayer and induces significant curvature that relaxes as the NP approaches the bilayer midplane. The ligand density within the bilayer core also increases as MUS ligands cross the bilayer and additional ligand backbones are able to access the favorable hydrophobic environment. Finally, the sharp peaks in the ligand density in the lipid head group region reflects electrostatic attraction between the zwitterionic lipid head groups and the anionic MUS end groups, as well as the geometric confinement imposed by the barrier for moving charged end groups into the hydrophobic tail group region.

### Multiple driving forces favor flipping

**Fig 5:**
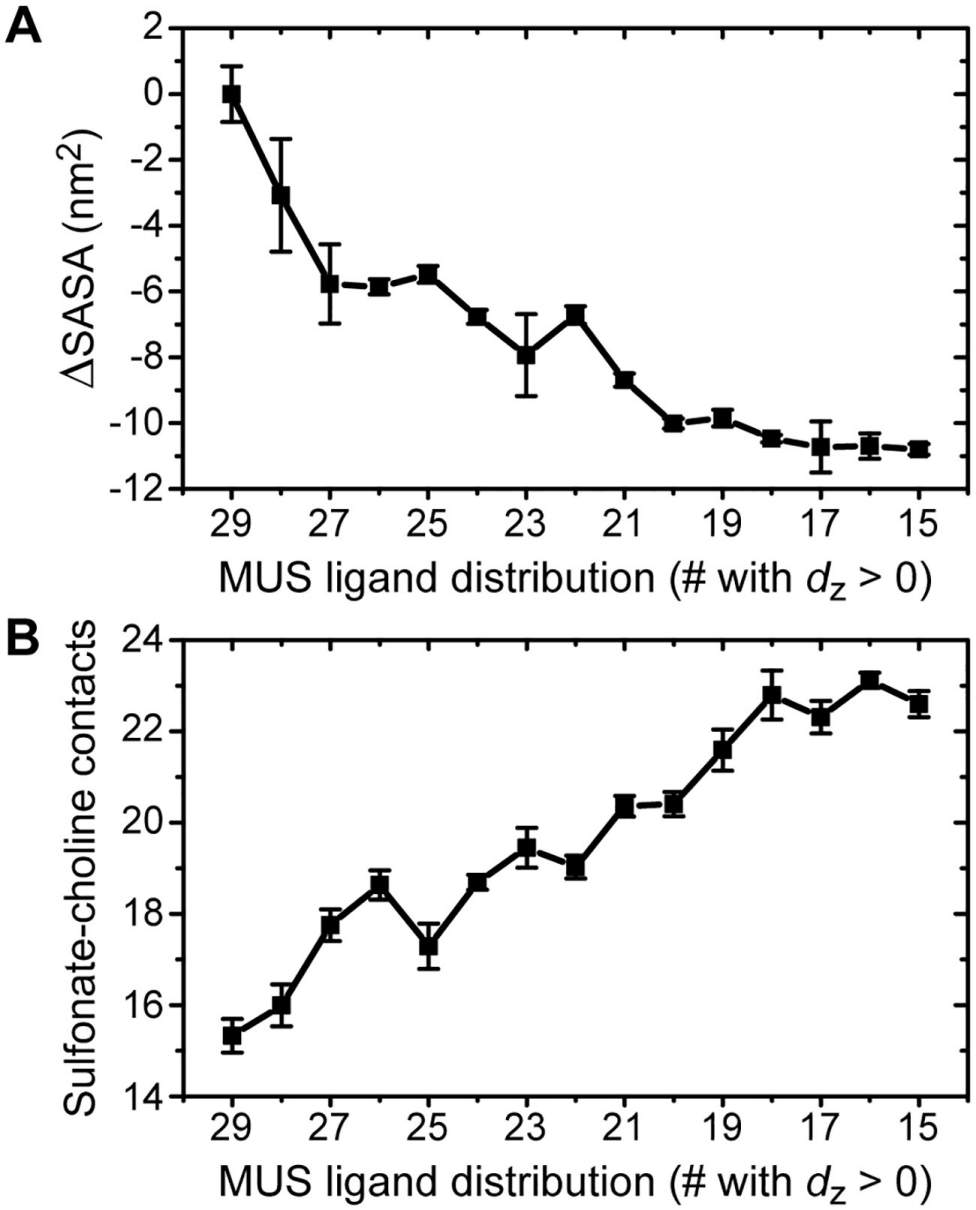
NP-bilayer interactions as a function of the distribution of MUS ligands. A: Decrease in the nonpolar solvent-accessible surface area of the NP ligands. B: Increase in the number of sulfonate-choline contacts. For both plots, error bars are computed using block averaging; error bars not visible are smaller than the symbols.

Prior work suggests that a fully inserted NP configuration is thermodynamically preferred compared to a partially inserted configuration (S1 Fig) [16, 29]. The density profiles in Fig 4 and analysis of the simulation trajectories point to three driving forces that favor the fully inserted configuration: (*i*) a decrease in the elastic bending energy of the membrane as bilayer curvature relaxes [61], (*ii*) a decrease in the unfavorable solvation free energy as hydrophobic ligand backbones enter the bilayer core [62], and (*iii*) an increase in electrostatic attraction as additional sulfonate groups contact zwitterionic lipid head groups. Fig 5 quantifies the latter two contributions, while the reduction in bilayer curvature is apparent in Fig 4.

Fig 5A shows the change in the average nonpolar solvent-accessible surface area (SASA) of the NP as MUS ligands flip across the bilayer. The SASA is calculated via the algorithm of Eisenhaber et al. [63] using the *g_sas* program of Gromacs with a probe radius of 0.14 nm. The change in SASA is calculated relative to the 29+/0- configuration. The SASA decreases as hydrophobic ligand backbones are sequestered within the bilayer core until plateauing when the NP reaches the 17+/12- configuration near the bilayer midplane (Fig 3). The maximum decrease in the SASA is 11.1 ± 0.6 nm^2^. For comparison, previous calculations indicate that the SASA decreases by 28.3 ± 2.1 nm^2^ when the NP first inserts into the upper leaflet of the bilayer from solution [29]. The change in the SASA can be approximately related to a corresponding hydrophobic driving force, Δ*G*_phob_, by the relation Δ*G*_phob_ = *γ*ΔSASA. *γ* is a solvation parameter that has been estimated as *γ* = 4.7 kcal/mol/nm^2^ for transferring hydrophobic solutes from water to an alkane environment [64]. Using this value, the large decrease in the SASA upon flipping corresponds to Δ*G*_phob_ = −52.1 ± 2.9 kcal/mol, and the near-monotonic decrease in the SASA suggests that each individual flip is driven by ligand hydrophobicity.

Fig 5B shows the average number of anionic sulfonate end groups in contact with cationic choline moieties in the DOPC head groups. A contact is counted if the distance between the sulfur atom in the sulfonate group and the nitrogen atom in the choline group is less than 0.7 nm. This threshold distance is equal to the position of the first minimum of the radial distribution function calculated for choline relative to a central sulfonate group (see S1 Text and S1 Table). As MUS ligands flip across the bilayer, the number of sulfonate-choline contacts increases, reflecting an increase in attractive electrostatic interactions between these species. As the initial adsorption of the NP to the bilayer surface is similarly due to electrostatic attraction [17, 28], the increase in sulfonate-choline contacts reflects a favorable electrostatic driving force for MUS flipping. The attraction between sulfonate and choline groups observed here agrees again with previous studies in which lipid head groups around a fully inserted NP reorient to maximize sulfonate-choline contacts [50].

In previous work, we found that the decrease in the SASA is the primary thermodynamic driving force that favors NP-bilayer insertion [8, 48]. These results show that all of the identified driving forces favor a membrane-spanning configuration, as flipping ligands decreases the SASA, relaxes membrane curvature, and increases favorable electrostatic interactions. While we did not quantify changes in ligand entropy, this effect is likely to be small relative to these other driving forces [8, 48]. Together, these findings strongly indicate that a membrane-spanning configuration is thermodynamically preferred relative to an asymmetric MUS ligand distribution.

### Dehydrated ligand end groups suggest rapid timescale for flipping

The results above identify multiple driving forces that favor a NP configuration in which MUS ligands are equally split on either side of the bilayer and the NP core sits at approximately the bilayer midplane. However, the flipping of each charged MUS end group is associated with a large energy barrier, one that may be too large for such an iterative flipping process to occur on experimentally relevant timescales. In recent work, we showed that the barrier for flipping a charged MUS ligand from the 15+/14- to 14+/15- configuration is nearly 7 kcal/mol lower than the barrier for translocating an isolated ion, allowing flipping to occur on a timescale of seconds [36]. The typical free energy barrier for translocating a charged species across a bilayer is chiefly due to two contributions: the free energy cost for partially dehydrating the ion’s solvation shell, and the free energy cost for deforming the bilayer to maintain partial ion solvation [36, 65]. The free energy barrier for flipping a ligand grafted to a NP is lower than the barrier for translocating an isolated ion because the grafted ligand is constrained near the bilayer-water interface and cannot access the bulk aqueous environment, inducing dehydration of its solvation shell prior to flipping [36]. As such, the free energy cost for flipping is solely due to lipid bilayer deformations.

**Fig 6:**
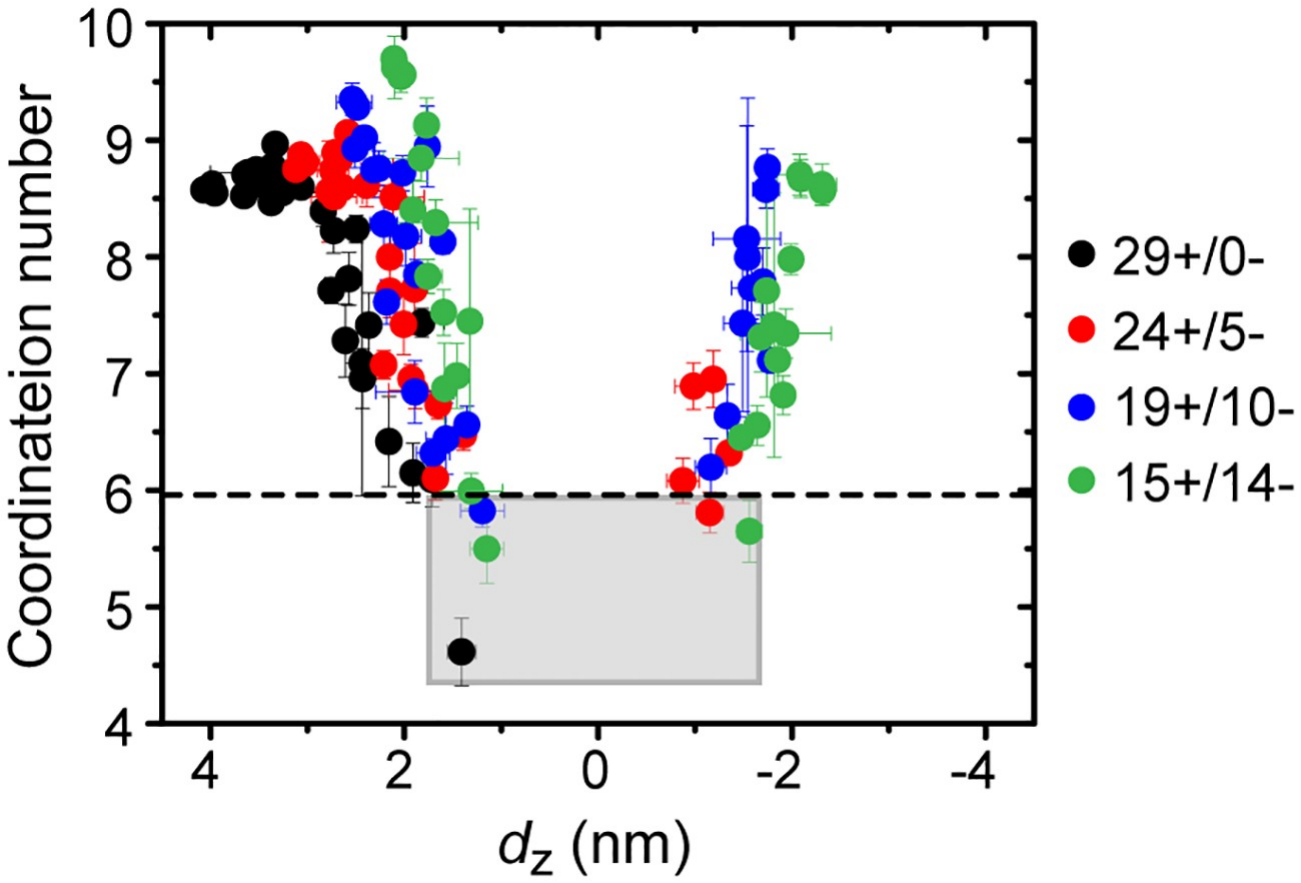
MUS end group coordination numbers as a function of the distance of each ligand from the bilayer midplane (*d*_*z*_). The average coordination number and average value of *d*_*z*_ is plotted for each of the 29 MUS end groups for four ligand distributions. Error bars are computed using block averaging; the average error in *d*_*z*_ is < 0.15 nm and the average error in the coordination number is < 0.25. The dashed line indicates the average coordination number of a ligand for which a reduced free energy barrier for flipping was calculated in prior work [36]. The points lying in the gray box correspond to ligands that are expected to flip on a timescale of seconds or less.

The free energy barrier for flipping was only calculated for a single distribution of MUS ligands due to the computational expense of these calculations. To determine if a similar free energy barrier would be expected for other MUS ligand distributions, Fig 6 presents the time-averaged coordination number of each MUS end group for four representative MUS ligand distributions. The coordination number is defined as the number of polar groups that have central atoms within a threshold distance equal to the position of the first minimum of the radial distribution function corresponding to each group (see S1 Text and S1 Table). The coordination number for each end group is plotted as a function of *d*_*z*_. The dashed horizontal line indicates the average value of the coordination number for the ligand previously found to exhibit a low flipping barrier [36]. As expected, the coordination number increases on average for end groups located farther from the bilayer midplane (where *d*_*z*_ = 0), illustrating that such end groups have greater access to water. For every MUS distribution, there is at least one end group for which *d*_*z*_ > 0.0 and the coordination number is equal to or lower than the average value of the coordination number that corresponds to a low flipping free energy barrier (corresponding to points lying within the gray box in Fig 6). This finding suggests that there is at least one ligand for every MUS distribution that can flip across the bilayer on experimentally relevant timescales due to its reduced coordination shell, generalizing prior results and supporting a pathway in which iterative ligand flipping enables the NP to reach a membrane-spanning configuration.

## Discussion

### Energy landscape for NP-bilayer insertion

**Fig 7:**
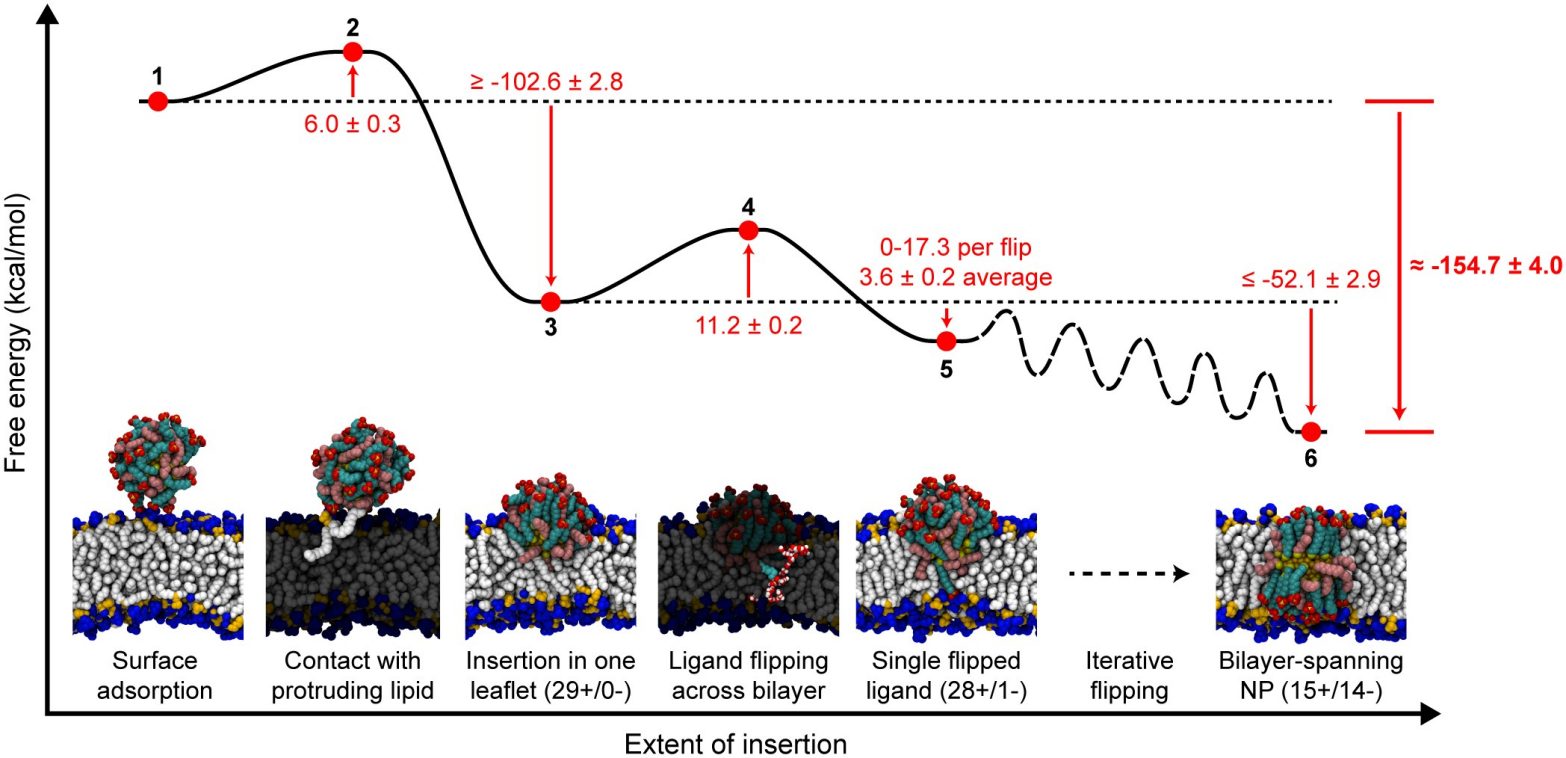
Energy landscape and simulation snapshots of representative configurations along the bilayer insertion pathway. All labeled values are reported in kcal/mol and the energy landscape is not drawn to scale We further note that the landscape is estimated subject to the assumptions discussed in the text. The free energy landscape does not include all possible ligand distributions which would be observed between states 5 and 6; the path between these states is labeled as “iterative flipping” to refer to the intermediate transitions between missing ligand distributions. The total free energy change between the initial (1) and final (6) states is highlighted in bold.

Building upon previous work [16, 29, 36, 46], the results presented here support a complete energy landscape for the insertion of amphiphilic monolayer-protected NPs into lipid bilayers. Fig 7 summarizes the insertion pathway with simulation snapshots of key intermediate steps. The NP first adsorbs to the surface of the bilayer (1) due to electrostatic interactions between the charged ligand end groups and dipolar lipid head groups [16, 17]. The NP then diffuses along the bilayer surface until encountering a solvent-exposed lipid tail protrusion (2). Contact with the tail protrusion triggers insertion into the upper leaflet of the bilayer to obtain the 29+/0- configuration (3). From this partially inserted configuration, charged end groups iteratively flip across the bilayer (barrier indicated by 4 and local minimum indicated by 5; additional configurations not shown) until a thermodynamically favorable membrane-spanning configuration is reached (6). This proposed pathway thus connects two stable states—a NP in solution near the bilayer surface (1) and a NP fully inserted into the bilayer (6)—which have been previously identified [8, 48] and provides additional insight into the intermediate states sampled during this transition that have not been studied in previous work [16, 28, 29, 36, 48]. The pathway also does not involve the formation of large bilayer defects, which is consistent with experimental observations of NPs entering both multilamellar vesicles and cells without allowing the passage of a membrane-impermeable dye [7, 8]. During ligand flipping, transient water defects do form in the bilayer to maintain a solvation shell around the charged MUS end group as previously reported [36]. However, these water defects dissipate on nanosecond timescales, and thus we assume that they would not facilitate dye leakage, unlike a stable membrane pore.

The free energy barriers and local free energy minima along the insertion pathway are also approximated in Fig 7 based on prior work and the present analysis of different ligand configurations with respect to the membrane. Here, we provide rationale for the estimated free energy of each state labeled in Fig 7. The free energy cost for a lipid tail protrusion (2) has been calculated as approximately 6.0 kcal/mol depending on the distance the tail protrudes into solution and the curvature of the bilayer [46]. The free energy change for the insertion of the NP into the 29+/0- configuration (3) can be estimated using a prior implicit solvent model that was found to correctly predict experimentally observed size thresholds for NP-bilayer insertion [8]. This model calculates the two-state free energy change between states (1) and (6), and thus assumes that the NP obtains a membrane-spanning configuration equivalent to state (6) [8, 48]. The free energy change for the asymmetric 29+/0- configuration is instead approximated as one-half the free energy change for the insertion of a larger NP that has approximately twice the number of ligands. From prior results, a 3 nm 1:1 MUS:OT NP with 130 surface ligands has a free energy change of approximately -232.1 ± 5.6 kcal/mol for inserting into a membrane-spanning configuration [8], or -115.05 ± 2.8 kcal/mol for inserting a 65 ligand NP into a single leaflet. Scaling this value to the 58 ligand NP studied here therefore yields an estimate of -102.6 ± 2.8 kcal/mol for insertion into the 29+/0- configuration. This value is lower than the free energy from the hydrophobic driving force alone, which is approximately -133.0 kcal/mol based on the 28.3 nm^2^ decrease in the SASA calculated previously [29]. However, insertion into a single leaflet induces significant bilayer deformations which strongly reduces ligand conformational freedom; therefore, the -102.6 kcal/mol estimate is reasonable and may be an overestimate. The barrier for each ligand flip (4) is approximately 11.2 ± 0.2 kcal/mol based on previous estimates [36] and should be similar for any ligand distribution based on the results in Fig 6. With each flip, the SASA decreases further, providing a driving force between 0-17.3 kcal/mol based on the results of Fig 5, with an average value of 3.6 ± 0.4 kcal/mol per flip. Assuming that the hydrophobic driving force is the dominant contribution to the overall free energy change, the free energy decreases by a total of -52.1 ± 2.9 kcal/mol during the iterative flipping pathway. This value is likely an underestimate because the relaxation of the bilayer and favorable electrostatic interactions further favor the iterative flipping process.

Based on these estimates, the total free energy change to reach a membrane-spanning configuration (6) is approximately -154.7 kcal/mol. This value is in good agreement with previous calculations that approximated the free energy change between states (1) and (6) as ≈ -150 kcal/mol [8] for a 2 nm 1:1 MUS:OT NP. For comparison, a purely hydrophobic NP with a diameter of 4 nm (approximately the diameter of the NPs studied here if the ligand shell is included [49]) would have a free energy change of ≈ -236 kcal/mol based on the SASA calculation. Similarly, the unfolding free energy of multispanning membrane proteins has been estimated as 290.5 to 485.5 kcal/mol [66]. These comparisons thus indicate that the inclusion of charged ligands do lower the stability of the MUS:OT NPs relative to purely hydrophobic NPs, but the order of magnitude for the insertion free energy is similar to highly stable membrane-embedded proteins.

We emphasize that the landscape presented in Fig 7 is estimated subject to the approximations described above. In principle, more accurate free energy estimates could be obtained from enhanced sampling techniques by performing umbrella sampling for each ligand flipping process to compute fourteen distinct potentials of mean force (PMFs) or by using techniques like metadynamics to fully sample a range of ligand distributions [36, 67]. However, these techniques are very computationally expensive—for example, calculating a single PMF for flipping a charged ligand across the bilayer using umbrella sampling requires approximately 60 ns of sampling for each of 60 umbrella sampling windows based on previous work [36]. Computing fourteen PMFs would thus require on the order of 50 *μ*s of simulation time; we leave such calculations for future work. Moreover, given the good agreement between the estimated free energy in this work and the two-state free energy change computed in past work, which was shown to predict experimental behavior [8], we expect that such calculations may not provide significant new physical insight to justify their computational expense.

### Alternative simulation methods and pathways

The proposed ligand flipping pathway is supported by atomistic molecular dynamics simulations, in which ligand degrees of freedom are explicitly modeled and electrostatic interactions are captured in detail. Comparing this pathway to results using other simulation methodologies emphasizes the importance of both of these features. Two recent studies of amphiphilic NP-bilayer interactions that employed the MARTINI coarse-grained force field, in which ligand degrees of freedom were explicitly included, identified charged ligand deformations that stabilize an inserted state in agreement with this work [27, 28]. In particular, the recent study by Simonelli et al. identified a similar pathway for bilayer insertion that also involves charged ligands flipping across the bilayer on microsecond timescales [28], although in these simulations progression to a fully inserted state is not observed within the simulation timescale. While the authors did not outline the thermodynamic factors that drive the insertion process and the reported flipping barrier (5-10 kcal/mol) is likely underestimated [36, 67], their results are in strong qualitative agreement with this study.

One of the important physicochemical features of the MUS:OT NPs studied here is the large surface charge of the NP monolayer. Prior atomistic molecular dynamics simulations have found that charged ligand end groups do not come into direct contact with the hydrophobic bilayer core during translocation [36, 67], instead forming water defects to facilitate translocation. Conversely, coarse-grained (CG) models that omit ligand degrees of freedom (e.g., by treating the NP as uniformly spherical) or explicit electrostatic interactions (e.g., by using short-range effective pair potentials between charged beads) predict qualitatively different behavior [31–33]. Notably, these techniques may lead to configurations in which several charged coarse-grained beads are in direct contact with the bilayer core, which may trigger significant bilayer disruption and poration [31] in contrast to the results presented here. An explicit description of NP surface ligands and electrostatic interactions thus appears to be necessary to model the insertion pathway described in this work. Nonetheless, these CG approaches can be powerful for systems that feature hydrophilic, but not highly charged, surface ligands, as these moieties face much smaller barriers for exposure to the bilayer core. For example, dissipative particle dynamics (DPD) simulations have been highly valuable for predicting NP-bilayer interactions for similar monolayer-protected NPs [23, 33], other nanomaterials [68], and for NPs coated in polymers [69]. Recent work using a simplifed version of the MARTINI CG force field has shown that tuning the hydrophobicity of a NP surface can also lead to near barrierless translocation across membranes [34]. Developing CG models that reproduce the findings from the atomistic simulations presented here will be explored in future work to further extend these findings to a larger range of NP surface compositions.

### Factors affecting flipping pathway

The simulation results presented here indicate that flipping is driven by the relaxation of bilayer curvature, lipid-ligand electrostatic interactions, and the decreased exposure of hydrophobic ligand backbones. These findings suggest several system features that may affect the thermodynamics of flipping. For example, tailoring NPs to increase the induced curvature, via incorporating longer hydrophobic ligands or patterning the surface in a Janus morphology, would favor a partially inserted state and possibly induce large scale membrane remodeling [61]. Similarly, membranes containing lipids with negative intrinsic curvatures may be recruited to the site of NP insertion to stabilize the partially inserted state [70]. Electrostatic interactions between ligands and lipids may also favor the recruitment of lipids with particular head groups, or may affect flipping in membranes with asymmetric lipid compositions [18]. Incorporating more hydrophobic ligands into the NP monolayer would increase the driving force for flipping to favor the fully inserted state, as has been suggested in prior calculations [48]. Finally, the length of the charged ligands could affect both the thermodynamics driving insertion and the energy barriers for flipping; while prior work suggests that shorter charged ligands decrease the driving force for insertion [48], the effect of ligand length on flipping energy barriers has yet to be explored.

### Relevance to cell uptake

Experimentally, the MUS:OT NPs studied in this work have been observed to enter cells via a non-endocytic, non-disruptive uptake mechanism [7], suggesting that MUS:OT NPs translocate directly across the cell membrane. The energy landscape for bilayer insertion supported by this work shows that MUS:OT NPs can flip charged ligands iteratively across the bilayer without introducing large bilayer defects that would be associated with bilayer disruption. Since bilayer disruption is linked to cytotoxicity [7], which must be carefully considered as an outcome when optimizing nanomaterials for biomedical applications [71], the non-disruptive pathway could enable efficient cytosolic access for drug delivery applications. However, the deep free energy minimum calculated for inserting the NP into the bilayer core suggests that MUS:OT NPs should preferentially reside within cell membranes rather than entering cells. To reconcile these computational and experimental findings, it is important to emphasize that the membranes considered in these simulations are single-component lipid bilayers that lack the complexity of biological membranes. Indeed, MUS:OT NPs do embed within model single-component lipid bilayers in agreement with the simulation predictions, as has been shown in experiments using black lipid membranes [8, 15], lipid vesicles [8, 14], and supported lipid bilayers [16]. Moreover, our prior work found that insertion into a model membrane is correlated with cellular internalization [8]. Several features of the cellular environment could facilitate cell entry. For example, thiol species present in the cytoplasm may trigger the removal of ligands from the NP monolayer [72], potentially triggering desorption from the membrane. Similarly, asymmetries in lipid composition between bilayer leaflets (particularly of anionic lipids) could affect the energy landscape presented here. The cell membrane is also regularly recycled by the cell, which could lead to internalization of membrane-embedded NPs. Finally, the cell interior contains membrane-bound compartments, so it is possible that NPs could transfer directly from the plasma membrane to membranes inside the cell. Thus, while there is still work to be done in clarifying translocation mechanisms in biological systems, this study helps understand interactions with model membranes; introducing biologically relevant asymmetries will be a focus of future work.

## Conclusion

In this work, we use atomistic molecular dynamics simulations to test the hypothesis that an amphiphilic, mixed-monolayer-protected gold nanoparticle reaches a membrane-spanning configuration by flipping charged MUS ligands across the bilayer midplane. First, ligands are iteratively flipped by biasing the dynamics of a single ligand end group. Each distribution of MUS ligands with respect to the bilayer is metastable over the 170 ns simulation time, and flipping does not lead to large bilayer pores or significantly increase the bilayer permeability. Flipping ligands is found to favor a fully inserted configuration by reducing the bilayer curvature, decreasing the amount of solvent-exposed nonpolar surface area, and increasing attractive lipid-ligand electrostatic interactions. It is further shown that at least one ligand has a partially dehydrated end group for several MUS distributions, suggesting that at least one ligand has a low free energy barrier for flipping for any MUS distribution [36]. These results support the iterative flipping pathway and are in strong qualitative agreement with recent coarse-grained simulations [28] while sampling all ligand distributions with respect to the bilayer. With these results, a complete pathway and corresponding energy landscape for NP insertion is described. In this pathway, a NP adsorbs to the bilayer interface, partially inserts into a single bilayer leaflet after contact with a solvent-exposed lipid tail protrusion, and iteratively flips single charged ligands across the bilayer until a symmetric ligand distribution is obtained.

The results presented here and the pathway described above are in good agreement with the experimental finding that amphiphilic NPs insert into cell membranes without allowing the passage of a membrane impermeable dye [7, 8]. This non-disruptive bilayer insertion pathway may explain the behavior of other NPs, as the primary requirements for this pathway are a hydrophobic driving force and sufficient ligand flexibility to avoid the exposure of charges to the bilayer core. Notably, recent experimental studies of similar monolayer-protected NP systems have also observed behavior consistent with bilayer insertion [73–75]. This work thus provides detailed physical insight into mechanism that will be valuable for designing NPs to maximize bilayer insertion that may generalize to a wide variety of nanomaterials.

## Supporting information

S1 Text Additional methodological details. (PDF)

S1 Fig Configurations from two previous simulations of the insertion of a 1:1 MUS:OT NP into a bilayer ribbon. At top, the simulation snapshots show starting configurations with all system components drawn (including water and ions); at bottom, the snapshots show final configurations with the MUS ligands highlighted. **A** A NP that inserts into the bilayer via the highly curved ribbon edge, representative of a large bilayer defect, distributes MUS end groups between both bilayer leaflets. **B** A NP that inserts into the planar face of a lipid ribbon, representative of insertion into a defect-free bilayer, retains all end groups on one side of the bilayer.
(TIF)

S2 Fig Details on workflow for flipping ligands to achieve different MUS distributions in NP-ribbon system. **A** Starting configuration for the 29+/0- MUS distribution with MUS end groups highlighted. *d*_*z*_, the distance between each end group and the bilayer midplane, is indicated; all 29 end groups have *d*_*z*_ > 0. The end group with the smallest value of *d*_*z*_ is circled. **B** Snapshots of the end group circled in A as it is flipped across the bilayer to generate the 28+/1- configuration. **C** *d*_*z*_ as a function of time for the five end groups with the smallest average values of *d*_*z*_ in the 29+/0- distribution. During the 20 ns, a single end group with a smallest average positive value of *d*_*z*_ can be identified. **D** Number of lipids within a 8.2 nm × 8.2 nm square centered on the NP for two different MUS distributions; the bounds of the square are illustrated as red lines in A. The number of lipids is counted in the upper and lower leaflets separately and rapidly equilibrates in each leaflet. In general, the two leaflets will have different numbers of lipids due to the difference in volume excluded by the NP.
(TIF)

S3 Fig Protocol for converting ribbon configurations to box-spanning bilayer configurations to reduce the total system size. **A** The NP and lipids within a 8.2 nm × 8.2 nm square (drawn in red) are extracted from the NP-ribbon system, then embedded within a larger bilayer from which a 8.5 nm × 8.5 nm square of lipids has been removed. The latter system is then equilibrated and used for additional sampling. This procedure reduces the system size while still increasing the bilayer dimensions due to the removal of excess solvent. Note that some lipids preferentially intercalate within the NP monolayer as observed previously. **B** Illustration of the extraction procedure from a side view, including both the original NP-ribbon system and resulting box-spanning NP-bilayer system. The box-spanning systems are used to compute all quantities in the main text.
(TIF)

S4 Fig Plots of observables calculated during 120 ns of unbiased sampling for three example MUS distributions. The distance of the NP to the bilayer midplane, the nonpolar SASA of the NP, and the number of sulfonate-choline contacts are presented; time-averaged values of these quantities are shown in the main text for all ligand distributions. No significant drift is observed in any quantity during the sampling time, confirming that the equilibration time is sufficient.
(TIF)

S1 Table Threshold distances used for calculating the number of sulfonate-choline contacts and the coordination number. (PDF)
