# Supplementary material for: Energy landscape for the insertion of amphiphilic nanoparticles into lipid membranes: A computational study
Source: PLoS One. 2019 Jan 9;14(1):e0209492. doi: 10.1371/journal.pone.0209492 (PMC6326551; doi:10.1371/journal.pone.0209492)
Supplement: S1 Text — (PDF) [file pone.0209492.s001.pdf]

Energy Landscape for the Insertion of Amphiphilic Nanoparticles into Lipid Membranes: A Computational Study

Reid C. Van Lehn* **and Alfredo Alexander-Katz**

S1 Text

1 Description of two bilayer types

As described in the main text and detailed below, two types of bilayers are simulated in this work: bilayer ribbons and box-spanning bilayers (S1 Fig). A bilayer ribbon spans the simulation box along only one axis (in this case, the y-axis), and has two solvent-exposed edges along the perpendicular x-axis [1]. In contrast, a box-spanning bilayer, which is more conventionally simulated, spans the entire x-y plane of the simulation box. Bilayer ribbons have been used when simulating systems that require significant bilayer expansion or curvature [2–5], as the two solvent-exposed edges allow the ribbon to expand laterally and bend without being constrained by the periodic boundaries of the simulation box. Accordingly, NP insertion into a planar bilayer was modeled using a ribbon in previous work because insertion induces bilayer curvature [5]. The final configuration from one of these simulations is used as the starting configuration for the workflow in which MUS ligands are iteratively flipped across the bilayer, as described below. We emphasize that the bilayer ribbons are only used for initial system preparation, while all observables presented in the main text are calculated using box-spanning bilayers to avoid any artifacts due to the ribbon edges.

2 Details on MUS flipping workflow

The flipping workflow described in the main text is performed using a NP-ribbon system to allow lipids to redistribute between the upper and lower bilayer leaflet during flipping. To flip a MUS ligand across the bilayer, a harmonic potential is applied to restrain the center-of-mass of the sulfonate end group to a reference distance from the bilayer midplane, projected along the z-axis; this reference distance is denoted by d_z (as defined in the main text; see S2 Fig):

$$U_{\text{spring}}(t) = \frac{K}{2}\left[z(t) - d_z\right]^2 \tag{1}$$

Here, $U_{\text{spring}}(t)$ is the harmonic potential at time t and $z(t)$ is distance between the center-of-mass of the sulfonate end group and the bilayer midplane, projected along the z-axis, at time t. The spring constant for the harmonic potential, K, is set to 3000 kJ/mol/nm^2. d_z is decreased at a rate of 2 nm/ns for 1.5 ns to flip the ligand across the bilayer midplane. The "cylinder" pulling geometry in Gromacs 4.6.7 is used so that the distance is computed relative to lipids only within a cylinder with a radius of 2.0 nm aligned along the z-axis. We emphasize that the harmonic potential is only applied to induce ligand flipping across the bilayer and is not used to compute a potential of mean force associated with the flipping process; a potential of mean force for this process has beeen computed in past work [6]. The magnitude of the spring constant is thus chosen only to ensure that flipping is observed and does not influence any results in the manuscript. The pressure is controlled using an anisotropic barostat so that only the x- and z-axes are permitted to resize to maintain the structure of the ribbon [4,5]. Position restraints are applied to the head groups of two lipids in the lower leaflet to prevent the ribbon from rotating.

After 1.5 ns of pulling, the umbrella potential is removed and the NP-ribbon system is allowed to relax for another 20 ns. During this equilibration process, lipids can transfer between the upper and lower bilayer leaflets via the ribbon edges without overcoming the large barrier for lipid flip-flop [7]. This lipid exchange mimics the equilibration of the transbilayer lipid distribution in a physical system that has access to a lipid reservoir. Without this free exchange of lipids, the difference in the area per lipid between the two leaflets due to the area excluded by the NP could introduce unphysical stresses into the system [8]. To confirm that 20 ns is sufficient time to equilibrate the lipid distribution, the number of lipids in the upper and lower leaflets that are within an 8.2 nm $\times$ 8.2 nm square (where 8.2 nm is the dimension of the fixed y-axis) centered on the NP is counted and found to exhibit minimal drift (S4 Fig). S4 Fig also shows that the ligand with the smallest value of d_z can be identified within the 20 ns timescale.

The flipping workflow is performed 14 times to produce 15 distinct ligand distributions with respect to the bilayer (from 29+/0- to 15+/14- using the terminology defined in the main text). To minimize computational expense and to eliminate any possible artifacts due to the ribbon edges, each of these 15 NP-ribbon configurations is converted to a conventional box-spanning configuration prior to additional sampling. A box-spanning configuration is created by extracting the NP and lipids within a 8.2 nm $\times$ 8.2 nm square (illustrated in S3 Fig). The square is positioned such that the number of lipids extracted from each leaflet of the ribbon is equal to the time-averaged number of lipids in the same leaflet within the same square area (rounded to the nearest integer) calculated during the last 10 ns of equilibration (S4 Fig). This extraction process thus preserves the transbilayer lipid distribution that is obtained during equilibration. Additional lipids are then equally added to the upper and lower leaflets by embedding the extracted square within a previously equilibrated bilayer from which a 8.5 nm $\times$ 8.5 nm square of lipids is removed, to achieve a total of 334 lipids in the system (S3 Fig). This procedure creates a system large enough to avoid finite-size effects by ensuring that any bilayer deformation induced by the NP decays within the simulation box (based on previous work[9]). Finally, water molecules and ions are added to the system to achieve an electroneutral 150 mM NaCl concentration with at least 3 nm of separation between the top of the NP and the bottom of the bilayer across the periodic boundary. The total number of each system component is identical for all systems to facilitate comparisons between different MUS ligand distributions. This process reduces the system size from 167,620 atoms in the NP-ribbon configuration (400 lipids, 48,272 water molecules, 167 Na^+ ions, and 138 Cl^- ions) to 83,338 atoms in the NP-bilayer configuration (334 lipids, 21,418 water molecules, 89 Na^+ ions, and 60 Cl^-). The NP-bilayer systems are equilibrated for 50 ns, then simulated for another 120 ns of production. The pressure in the box-spanning bilayer system is maintained using a semiisotropic barostat. S4 Fig confirms that simulation observables do not drift during the 120 ns sampling time.

3 Model for the gold core and ligand monolayer

Following previous work[4,10], the GROMOS 54a7 united atom force field was used to model the lipids, ions, and NP in conjunction with the SPC water model[11]. Lennard-Jones (LJ) parameters for the sulfonate end groups used standard GROMOS values[12] while the partial charges were adapted from a parameterization of ionic liquids[13]. All sulfonate groups were assumed to be fully dissociated given the low pKa of sulfonate. LJ parameters for interactions between gold and alkane beads were taken from the Hautman-Klein model using a parameterization fit to the GROMOS 12-6 LJ form[14,15]. Similar treatments of the interactions between gold and hydrocarbons have been used in several previous studies[4,16–18]. All other LJ interactions with gold were calculated using the parameters for gold from the Universal Force Field and standard GROMOS combination rules[19]. However, in practice only gold-hydrocarbon interactions were relevant due to the steric barrier imposed by the protecting monolayer. Gold atoms were treated as uncharged and no polarization effects were included under the assumption that surface properties are dominated by the protecting monolayer.

The NP core was represented as a hollow, perfectly spherical gold shell with a 2.0 nm diameter. The mass of missing core atoms was redistributed to the surface atoms. Constraints were placed between near-neighbor gold atoms to maintain a rigid spherical structure. The sulfur head groups of the grafted monolayer were distributed uniformly across the surface at a grafting density of 4.62 ligands/nm^2 yielding 58 ligands. This grafting density corresponds to the density of alkanethiol ligands when grafted to the (111) surface of a planar gold lattice. Despite the high curvature of the small NP gold core, this grafting density is consistent with prior density functional theory calculations[18,20] and experimental estimates[21]. The sulfur head groups were rigidly grafted to the gold core using constraints to the nearest gold atoms, eliminating any diffusion of ligands on the surface. The two different ligand species (MUS and OT) were distributed in a "checkerboard"-like morphology as in previous work[4,10,22]. Our prior computational work has suggested that the spatial morphology of the two ligand types (*e.g.*, randomly distributed, checkerboard-like, or striped) does not affect NP properties, which are dominated by ligand properties alone[10]. Therefore, the simplified representation of the NP is appropriate for this system. Following the Hautman-Klein model, no bond angle or dihedral constraints were placed on the carbon atom bonded to the thiol group grafted to the NP surface allowing ligand flexibility at the gold interface[14].

One of the potential weaknesses of the model used in this work is the treatment of the gold core as a hollow shell of gold atoms because interactions with the missing gold atoms are omitted and any image charge interactions that could emerge from the polarizablity of the gold core are absent. We justify these omissions based on past work that has investigated the organization of alkanethiol ligands on gold nanoparticles with several assumptions regarding the treatment of the gold core. Notably, our results using the same hollow core model[10] yield radial distribution functions that are nearly indistinguishable from results for similar monolayer-protected NPs in which gold core charges are fit to reproduce DFT calculations and gold atoms are included explicitly[18]. Recent work using a force field that includes the polarizability of the gold core has also shown that interactions with

a bare gold core decay over an approximately nanometer length scale[23] that is similar to the thickness of the ligand monolayer, suggesting that interactions with the core itself likely to do not contribute to NP-bilayer interactions. Thus, while it is possible that including the gold core could have a minor effect on the system (as could a more accurate representation of the NP core shape), the interactions with the bilayer and surrounding environment are dominated by the ligands themselves and omitting more accurate interactions with gold is not expected to affect the results presented in the main text.

4 Calculation of coordination number

In the main text, the coordination number reports on the effective solvation shell of a sulfonate group and is defined as the number of polar groups within a threshold distance of the ion. In practice, the anionic sulfonate group recruits water molecules, cationic sodium ions, and cationic choline groups into its solvation shell. Because each of these species differs in size, a separate threshold distance is defined for each group by calculating the radial distribution function, $g(r)$, between the central sulfur atom in the sulfonate group and the central atom in each polar group (summarized in S1 Table). The radial distribution function for each of the three species is calculated from the 120 ns 15+/14- trajectory. The threshold distance is then defined as the distance corresponding to the first local minimum in $g(r)$. S1 Table summarizes the three different distance thresholds used to calculate the coordination number (Fig 6 of the main text). The distance threshold for choline is also used to calculate the number of sulfonate-choline contacts (Fig 5 of the main text).

References

1 A. West, K. Ma, J. L. Chung and J. T. Kindt, *J. Phys. Chem. A*, 2013, **117**, 7114–7123.
2 D. E. Chandler, J. Hsin, C. B. Harrison, J. Gumbart and K. Schulten, *Biophys. J.*, 2008, **95**, 2822–36.
3 A. Arkhipov, Y. Yin and K. Schulten, *Biophys. J.*, 2009, **97**, 2727–2735.
4 R. C. Van Lehn, M. Ricci, P. H. J. Silva, P. Andreozzi, J. Reguera, K. Voïtchovsky, F. Stellacci and A. Alexander-Katz, *Nat. Commun.*, 2014, **5**, 4482.
5 R. C. Van Lehn and A. Alexander-Katz, *Soft Matter*, 2015, **11**, 3165–3175.
6 R. C. Van Lehn and A. Alexander-Katz, *ACS Cent. Sci.*, 2017, **3**, 186–195.
7 D. P. Tieleman and S.-J. Marrink, *J. Am. Chem. Soc.*, 2006, **128**, 12462–12467.
8 S. Park, A. H. Beaven, J. B. Klauda and W. Im, *J. Chem. Theory Comput.*, 2015, **11**, 3466–3477.
9 R. C. Van Lehn and A. Alexander-Katz, *J. Phys. Chem. B*, 2014, **118**, 12586–12598.
10 R. C. Van Lehn and A. Alexander-Katz, *J. Phys. Chem. C*, 2013, **117**, 20104–20115.
11 N. Schmid, A. P. Eichenberger, A. Choutko, S. Riniker, M. Winger, A. E. Mark and W. F. van Gunsteren, *Eur. Biophys. J.*, 2011, **40**, 843–856.
12 M. J. Hinner, S.-J. J. Marrink and A. H. De Vries, *J. Phys. Chem. B*, 2009, **113**, 15807–19.
13 J. N. C. Lopes, A. A. H. Pádua and K. Shimizu, *J. Phys. Chem. B*, 2008, **112**, 5039–5046.
14 J. Hautman and M. L. Klein, *J. Chem. Phys*, 1989, **91**, 4994.
15 R. Mahaffy, R. Bhatia and B. J. Garrison, *J. Phys. Chem. B.*, 1997, **101**, 771–773.
16 P. Ghorai and S. Glotzer, *Journal of Physical Chemistry C*, 2007, **111**, 15857–15862.
17 O.-S. Lee and G. C. Schatz, *J. Phys. Chem. C*, 2009, **113**, 2316–2321.
18 E. Heikkilä, A. a. Gurtovenko, H. Martinez-Seara, H. Häkkinen, I. Vattulainen and J. Akola, *J. Phys. Chem. C*, 2012, **116**, 9805–9815.
19 A. K. Rappe, C. J. Casewit, K. S. Colwell, W. A. Goddard and W. M. Skiff, *J. Am. Chem. Soc.*, 1992, **114**, 10024–10035.
20 O. Lopez-Acevedo, J. Akola, R. L. Whetten, H. Gronbeck and H. Hakkinen, *J. Phys. Chem. C*, 2009, **144**, 5035–5038.
21 Y. Jiang, S. Huo, T. Mizuhara, R. Das, Y.-W. Lee, S. Hou, D. F. Moyano, B. Duncan, X.-j. Liang and V. M. Rotello, *ACS Nano*, 2015, **9**, 9986–9993.
22 R. C. Van Lehn, P. U. Atukorale, R. P. Carney, Y.-S. Yang, F. Stellacci, D. J. Irvine and A. Alexander-Katz, *Nano Lett.*, 2013, **13**, 4060–4067.
23 I. L. Geada, H. Ramezani-Dakhel, T. Jamil, M. Sulpizi and H. Heinz, *Nat. Commun.*, 2018, **9**, 716.
