# Supplementary figures and images for: Energy landscape for the insertion of amphiphilic nanoparticles into lipid membranes: A computational study
Source: PLoS One. 2019 Jan 9;14(1):e0209492. doi: 10.1371/journal.pone.0209492 (PMC6326551; doi:10.1371/journal.pone.0209492)

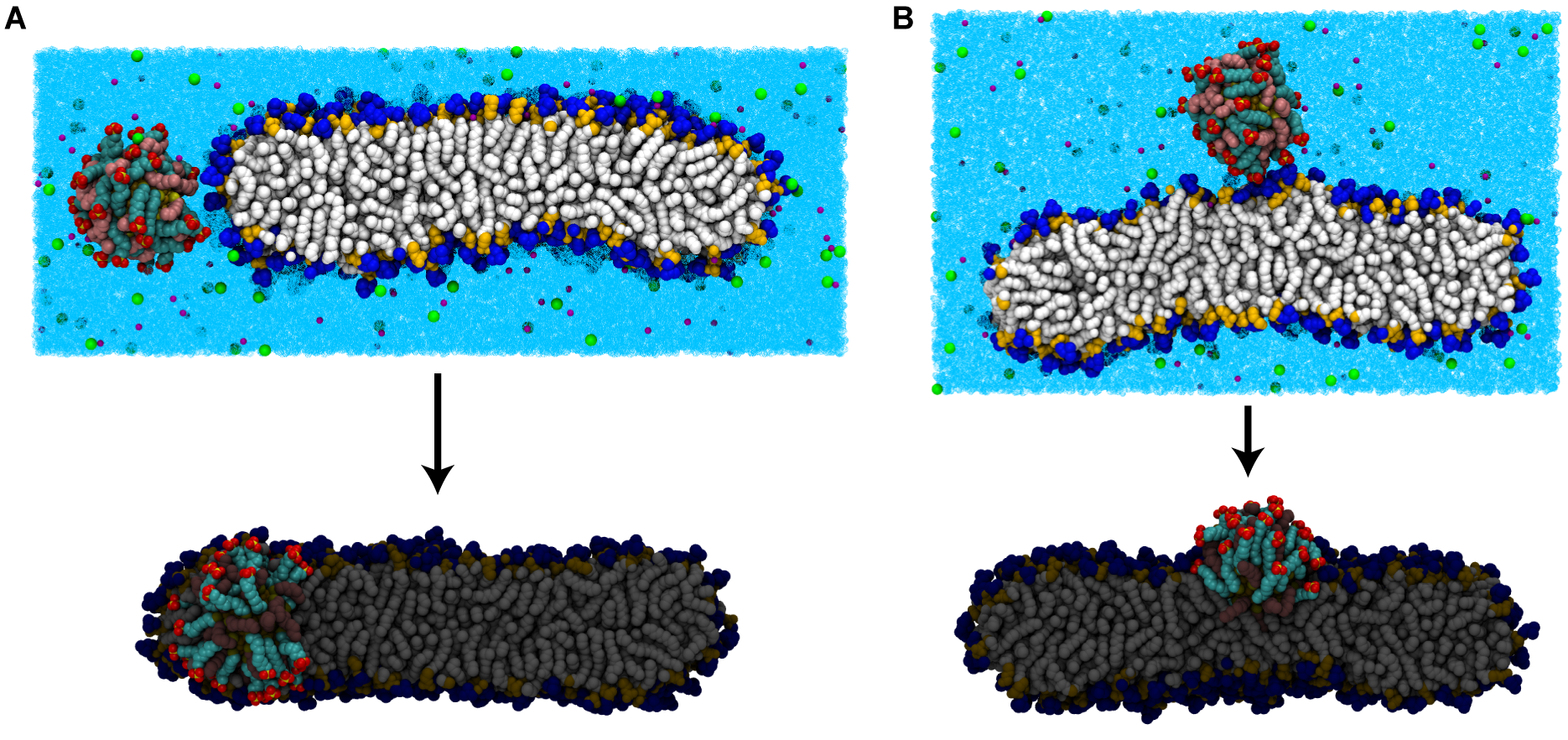

Supplement: S1 Fig — At top, the simulation snapshots show starting configurations with all system components drawn (including water and ions); at bottom, the snapshots show final configurations with the MUS ligands highlighted. A A NP that inserts into the bilayer via the highly curved ribbon edge, representative of a large bilayer defect, distributes MUS end groups between both bilayer leaflets. B A NP that inserts into the planar face of a lipid ribbon, representative of insertion into a defect-free bilayer, retains all end groups on one side of the bilayer. (TIF) [file pone.0209492.s002.tif]

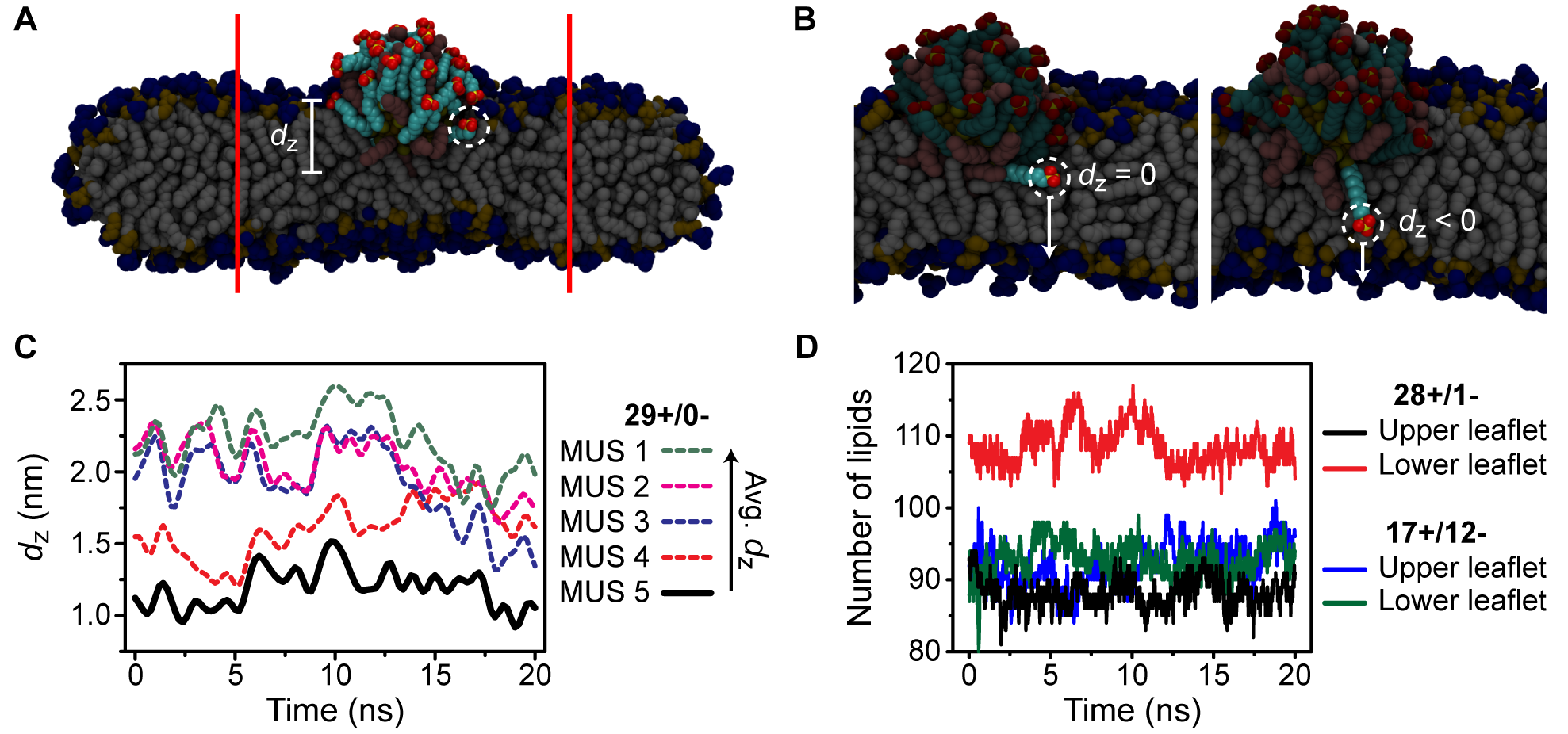

Supplement: S2 Fig — A Starting configuration for the 29+/0- MUS distribution with MUS end groups highlighted. dz, the distance between each end group and the bilayer midplane, is indicated; all 29 end groups have dz > 0. The end group with the smallest value of dz is circled. B Snapshots of the end group circled in A as it is flipped across the bilayer to generate the 28+/1- configuration. C dz as a function of time for the five end groups with the smallest average values of dz in the 29+/0- distribution. During the 20 ns, a single end group with a smallest average positive value of dz can be identified. D Number of lipids within a 8.2 nm × 8.2 nm square centered on the NP for two different MUS distributions; the bounds of the square are illustrated as red lines in A. The number of lipids is counted in the upper and lower leaflets separately and rapidly equilibrates in each leaflet. In general, the two leaflets will have different numbers of lipids due to the difference in volume excluded by the NP. (TIF) [file pone.0209492.s003.tif]

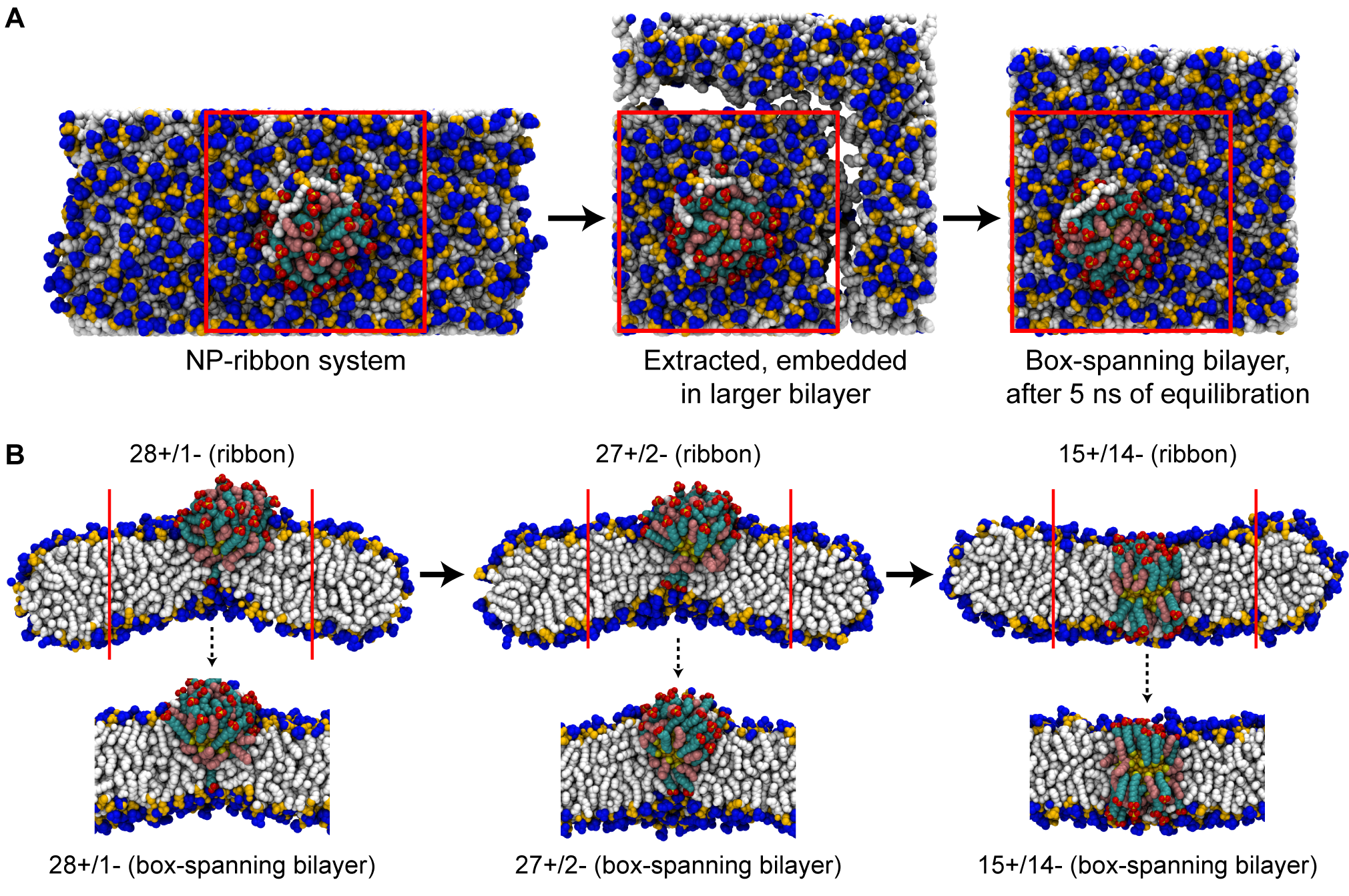

Supplement: S3 Fig — A The NP and lipids within a 8.2 nm × 8.2 nm square (drawn in red) are extracted from the NP-ribbon system, then embedded within a larger bilayer from which a 8.5 nm × 8.5 nm square of lipids has been removed. The latter system is then equilibrated and used for additional sampling. This procedure reduces the system size while still increasing the bilayer dimensions due to the removal of excess solvent. Note that some lipids preferentially intercalate within the NP monolayer as observed previously. B Illustration of the extraction procedure from a side view, including both the original NP-ribbon system and resulting box-spanning NP-bilayer system. The box-spanning systems are used to compute all quantities in the main text. (TIF) [file pone.0209492.s004.tif]

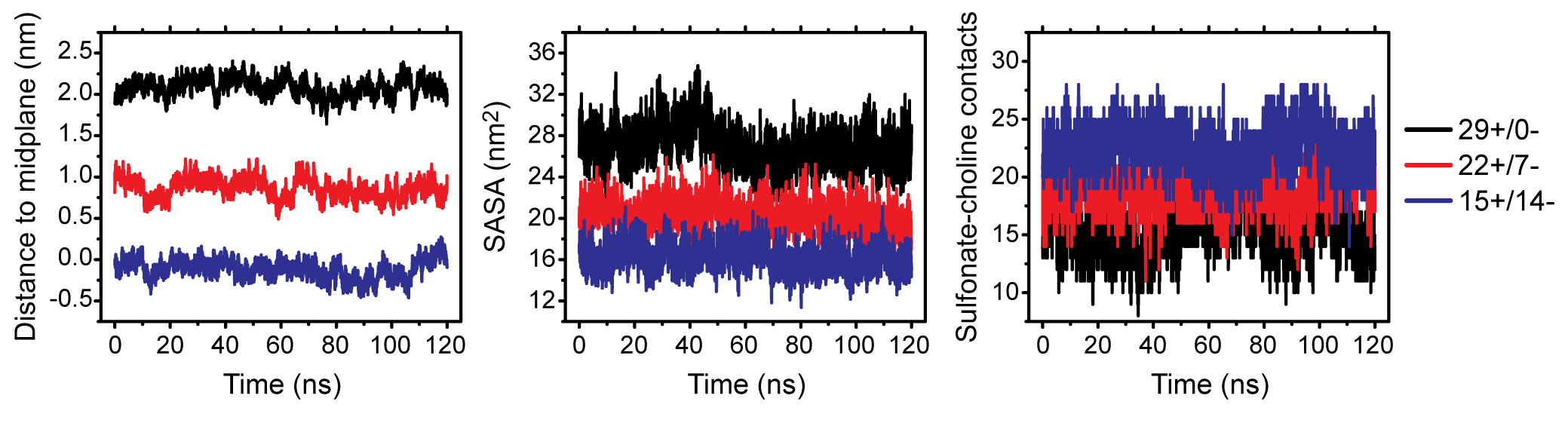

Supplement: S4 Fig — The distance of the NP to the bilayer midplane, the nonpolar SASA of the NP, and the number of sulfonate-choline contacts are presented; time-averaged values of these quantities are shown in the main text for all ligand distributions. No significant drift is observed in any quantity during the sampling time, confirming that the equilibration time is sufficient. (TIF) [file pone.0209492.s005.tif]
