# Supplementary material for: Energy landscape for the insertion of amphiphilic nanoparticles into lipid membranes: A computational study
Source: PLoS One. 2019 Jan 9;14(1):e0209492. doi: 10.1371/journal.pone.0209492 (PMC6326551; doi:10.1371/journal.pone.0209492)
Supplement: S1 Table — (PDF) [file pone.0209492.s006.pdf]

S1 Table Threshold distances used for calculating the number of sulfonate-choline contacts and the coordination number.

**Polar group:**	Water	Na^+	Choline
**Central atom:**	O	Na	N
**Threshold distance (nm):**	0.46	0.42	0.70
